# Obesity alters the collagen organization and mechanical properties of murine cartilage

**DOI:** 10.1038/s41598-020-80599-1

**Published:** 2021-01-15

**Authors:** Amber T. Collins, Guoli Hu, Hunter Newman, Michael H. Reinsvold, Monique R. Goldsmith, John N. Twomey-Kozak, Holly A. Leddy, Deepika Sharma, Leyao Shen, Louis E. DeFrate, Courtney M. Karner

**Affiliations:** 1 Department of Orthopaedic Surgery, Duke University School of Medicine, DUMC Box 3093, Durham, NC 27710 USA; 2 Department of Cell Biology, Duke University School of Medicine, Durham, NC 27710 USA; 3 Shared Materials Instrumentation Facility, Pratt School of Engineering, Duke University, Durham, NC 27710 USA; 4 Department of Biomedical Engineering, Pratt School of Engineering, Duke University, Durham, NC 27710 USA; 5 Department of Mechanical Engineering and Materials Science, Pratt School of Engineering, Duke University, Durham, NC 27710 USA; 6 Charles and Jane Pak Center for Mineral Metabolism and Clinical Research, Department of Internal Medicine, University of Texas Southwestern Medical Center, Dallas, TX 75390 USA

**Keywords:** Cartilage, Biomedical engineering, Applications of AFM, Obesity

## Abstract

Osteoarthritis is a debilitating disease characterized by cartilage degradation and altered cartilage mechanical properties. Furthermore, it is well established that obesity is a primary risk factor for osteoarthritis. The purpose of this study was to investigate the influence of obesity on the mechanical properties of murine knee cartilage. Two-month old wild type mice were fed either a normal diet or a high fat diet for 16 weeks. Atomic force microscopy-based nanoindentation was used to quantify the effective indentation modulus of medial femoral condyle cartilage. Osteoarthritis progression was graded using the OARSI system. Additionally, collagen organization was evaluated with picrosirius red staining imaged using polarized light microscopy. Significant differences between diet groups were assessed using *t* tests with p < 0.05. Following 16 weeks of a high fat diet, no significant differences in OARSI scoring were detected. However, we detected a significant difference in the effective indentation modulus between diet groups. The reduction in cartilage stiffness is likely the result of disrupted collagen organization in the superficial zone, as indicated by altered birefringence on polarized light microscopy. Collectively, these results suggest obesity is associated with changes in knee cartilage mechanical properties, which may be an early indicator of disease progression.

## Introduction

Osteoarthritis (OA) is a degenerative joint disease characterized by functional impairment due to pathological changes that include degradation of the articular cartilage, osteophyte formation, subchondral sclerosis, and synovitis^1,2^. The estimated lifetime risk of symptomatic knee OA is approximately 14% and can be as high as 24% in obese females^3^. As there are no known disease modifying drugs for OA, current management options are primarily limited to treatments such as exercise, nonsteroidal anti-inflammatory drugs, and pain medications^4^. In severe cases, clinicians recommend total joint replacement (TJR) for end-stage OA^5^. Several known risk factors exist for the development of knee OA including age, anatomical factors, past joint injury, and obesity. The association between obesity and OA has been well-established, as an elevated body mass index (BMI) ≥ 30 kg/m^2^ (the clinical definition of obesity)^6^ is associated with both inflammatory and degenerative conditions in the knee, increasing the need for TJR^7–9^. Considering obesity globally affects approximately 650 million adults^10^, there is heightened cause for concern. Despite the well-established relationship between obesity and OA, the exact mechanism of this association remains unclear.

Previous studies have established a relationship between body weight and OA in weight-bearing joints such as the knee^11–13^. To this point, a number of studies have hypothesized that altered loading in the knee joint predisposes cartilage to degeneration^14^. Recent studies have demonstrated increased cartilage strains in participants with high BMI^15,16^. Because it is difficult to directly evaluate cartilage mechanical properties in vivo, it is unclear whether these increased strains are due to elevated joint loading, decreased cartilage stiffness, or a combination of both.

As an alternative to in vivo testing, diet-induced obese mouse models have been used to study OA pathogenesis^17–20^ primarily due to their accelerated OA onset and progression. Using a mouse model provides the opportunity to directly evaluate changes in both histological and mechanical cartilage properties in the context of diet-induced obesity. However, conventional biomechanical testing in mice is challenging given the small size of the murine knee joint. Therefore, atomic force microscopy (AFM) can be used to evaluate the mechanical properties of murine cartilage tissue^21–24^.

Here, we used an integrative approach to define both the mechanical and compositional changes in murine cartilage due to diet-induced obesity. Based on prior work in human subjects^15^, we hypothesized that cartilage in obese mice would be less stiff compared to normal weight controls. Indeed, our results show that obese mice had decreased cartilage stiffness coincident with disruption of collagen organization in the superficial zone. Importantly, these changes preceded traditional histological measures of cartilage degeneration. Collectively, these findings indicate that mechanical changes are an early characteristic of OA development due to obesity.

## Methods

### Study design

Two-month old male mice (C57BL/6J) were fed ad libitum either a normal diet (ND, n = 10) containing 12.6% kCal from fat (Envigo TD.08485) or a high fat diet (HFD, n = 15) containing 42% kcal from fat (Envigo TD.88137) for a period of 16 weeks. The mice were weighed once a week. At the end of the 16 weeks, all mice were euthanized. The Animal Studies Committee at Duke University approved all mouse procedures. All additional methods were carried out in accordance with relevant guidelines and regulations.

### Glucose tolerance testing

At week 16, glucose levels were measured in blood obtained directly from the distal tail tip after removal of 1 mm of tissue using sharp scissors. Mice underwent a 6 h morning fast prior to determination of the fasting blood glucose levels. Mice were then given 2 g/kg glucose (Sigma G8769) intraperitoneally and blood glucose was measured 15, 30, 60, and 120 min after injection. Blood glucose was measured from approximately 1–3 µL of blood at each time point using a whole blood glucose meter (GLUCOCARD Vital).

### Sample preparation

Following euthanasia, hind limbs were harvested from all mice for AFM nanoindentation and histological analysis. The left hind limb of each mouse was harvested and wrapped in phosphate-buffered saline (PBS)-soaked gauze supplemented with a protease inhibitor cocktail (Roche 11697498001) and stored at − 20 °C for subsequent AFM testing. The right hind limbs were harvested and fixed for 72 h in 10% neutral buffered formalin, followed by a 2-week decalcification in 14% ethylenediaminetetraacetic acid (EDTA). They were then embedded in paraffin for histological analysis.

### Cartilage and liver histology

Paraffin-embedded joints were sectioned at 5 µm thickness, stained using Safranin-O and fast green, and graded for OA severity by four independent, blinded observers using the OsteoArthritis Research Society International (OARSI) scoring system^25^. Three sections (medial) from each joint were stained and evaluated. Sections were evaluated separately for the femur and tibia and averaged across joints. Adjacent sections were stained with Alcian blue and Picrosirius red and subsequently imaged under polarized light to visualize collagen fiber organization^26^. Birefringence of collagen fibers was then determined based on the intensity of individual pixels captured using polarized light microscopy^27^. To confirm effectiveness of the HFD, livers were harvested, fixed in 4% paraformaldehyde in PBS for 16 h at 4 °C, washed 3 times with PBS, and cryoprotected in 30% sucrose for 16 h at 4 °C. Specimens were then embedded in optimal cutting temperature compound (Sakura 4583) and cryosectioned at 10 µm thickness. Hematoxylin and eosin (H&E) or Oil Red O staining was performed according to standard protocols.

### Immunohistochemistry

After deparaffinization and rehydration, antigens were retrieved using proteinase K (10 ng/ml in PBS) at room temperature for 10 min. Sections were rinsed three times with PBS and incubated with DAKO dual endogenous enzyme blocking reagent (DAKO S2003) at room temperature for 30 min. The slides were rinsed three times with 0.1% Tween-20 in PBS (PBST) and then blocked with 2% normal goat serum at room temperature for 30 min. Next, slides were incubated at 4 °C overnight with antibodies raised against SOX-9 (Abcam, ab 26414, 1:200), Collagen-II (Thermo Scientific, MS235-P, 1:200), MMP13 (Thermo-fisher, MS-825-P, 1:100), or the Aggrecan C-terminal neoepitope NITEGE (MD Bioproducts, 1042003, 1:200). Sections were rinsed three times with PBST and incubated at room temperature for two hours with the appropriate secondary antibody (Vector Laboratories, PK-6105). Color reaction was performed using Vector ImmPact DAB kit (Vector Laboratories, SK-4105). Relative staining intensity and cell quantity (%) were quantified using Image J software (Image J V1.8.0).

### AFM-based nanoindentation

Prior to AFM analysis, joints were thawed overnight at 4 °C and the femur was disarticulated from the tibia. Muscle and ligaments were carefully removed from the distal end of the femur to protect the femoral cartilage. The resulting dissected distal femur was then mounted onto an AFM circular disk via cyanoacrylate glue. Samples were then covered in PBS in order to maintain tissue hydration. AFM-based nanoindentation was performed on the surface of each medial femoral condyle using a silicon nitride cantilever with a gold (Au) coated borosilicate spherical bead^28^ with a tip radius of 5 µm (Novascan). Each cantilever had a nominal spring constant (k) of approximately 0.6 N/m. Calibration of the cantilever deflection sensitivity (nm/V) was conducted using a hard calibration disk (Asylum Research). Exact spring constants were determined by thermal oscillation in PBS in order to maintain the same conditions as nanoindentation testing (Cypher AFM, Asylum Research). For each condyle, the cartilage was indented at 2–3 locations and at each location, nanoindentation was repeated six times. In determining the indentation locations on the medial femoral condyle, the cartilage surface was visually assessed via the microscope feature of the AFM setup to identify a relatively flat location for indentation. After multiple indentations were made at this location, the cantilever was disengaged and moved at least 50 µm in a different direction. The process was repeated until 3 different indentation locations were assessed. Each indentation was performed with a velocity of 1.98 µm/s and a maximum indentation force of approximately 30 nN. The loading portion of the approach curve was analyzed by the Hertzian contact equation, which models indentation of a rigid sphere into a semi-infinite elastic solid^29^. Specifically, an effective indentation modulus (E_ind_) was calculated by fitting the loading portion of each approach curve using a least squares regression based on previous work^30^:

$$F = \frac{4}{3}\frac{{E_{ind} }}{{(1 - \upsilon^{2} )}}R^{1/2} D^{3/2}$$

where F is the applied force, R is the tip radius (5 µm), υ is Poisson’s ratio (0.1 for murine cartilage^29,31^), and D is the indentation depth. The coefficient of determination was used to compare the goodness-of-fit for each force displacement curve. This measurement of an effective indentation modulus is useful for comparing moduli of different specimens under similar testing conditions, allowing for the identification of relative differences in mechanical behavior^21,22,24,29^.

### Statistical analysis

A sample size approximation was made a priori based on a previous study investigating the changes in mechanical properties of murine cartilage in a model of meniscus injury^22^. In that study, the authors demonstrated a significant reduction in cartilage modulus with 10 murine joints per group^22^. The presence of data outliers was tested using visual inspection of the inner and outer fences of the interquartile ranges, and normality was assessed by inspection of the kernel density plots of the residuals. Because 4 outliers were identified (2 for modulus and 2 for OARSI score), a sensitivity analysis was performed and it was determined that no statistical interpretations were altered by the presence of these outliers. Therefore, all data points were included for statistical analysis. Outcome measures between diet groups were compared using Student’s *t* test where a p value of less than 0.05 was considered statistically significant.

## Results

### Systemic changes caused by high fat diet feeding

**Figure 1:**
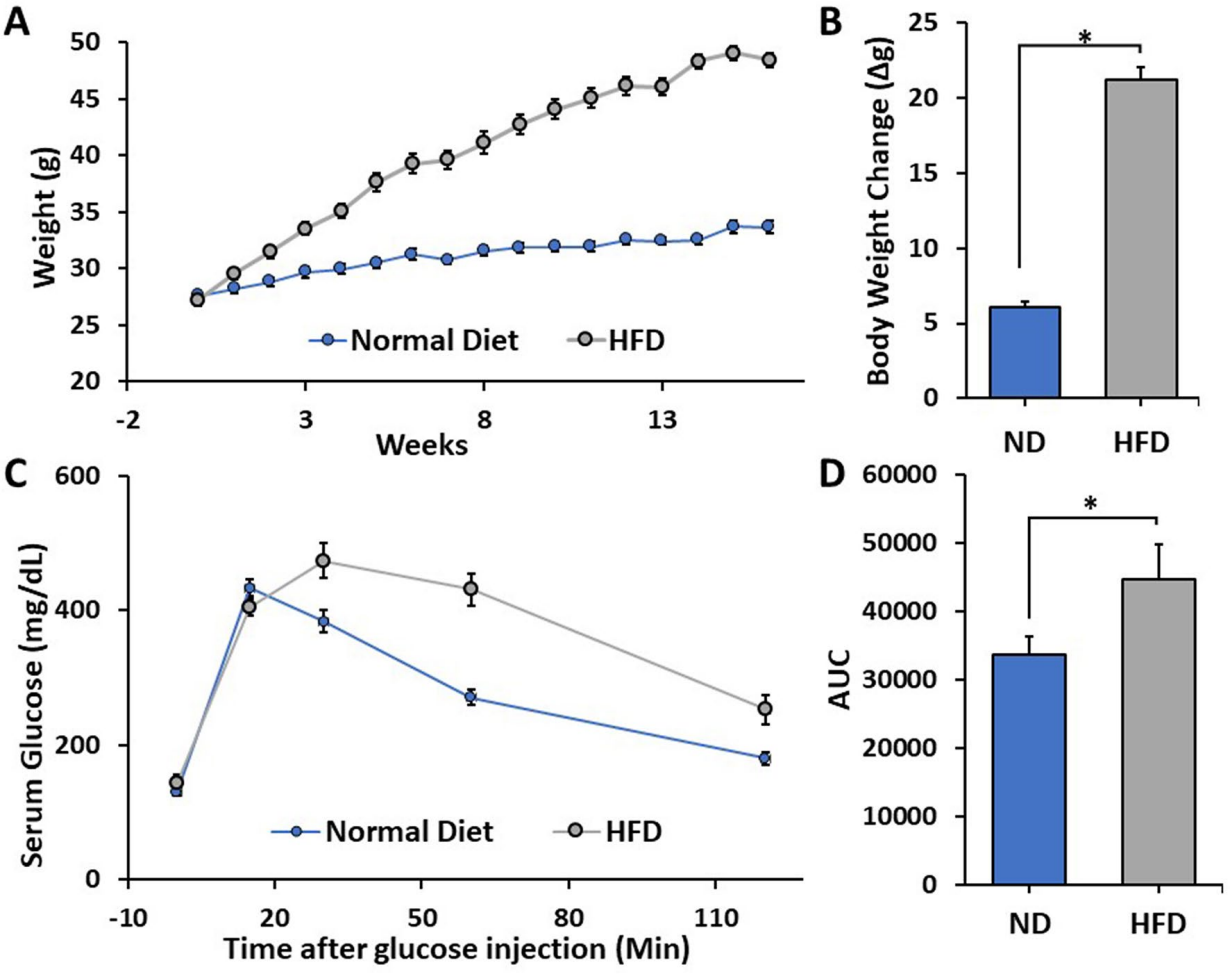
(**A**,**B**) Graphical representation of the absolute body weight (**A**) and increase in body weight (**B**) of mice fed either a normal chow diet (ND) or high fat diet (HFD) for 16 weeks. (**C**) Evaluation of serum glucose concentration following intraperitoneal glucose administration (2 g/kg). (**D**) Graphical depiction of area under curve (AUC) values in (**C**). Mean ± SEM. *p < 0.05.

**Figure 2:**
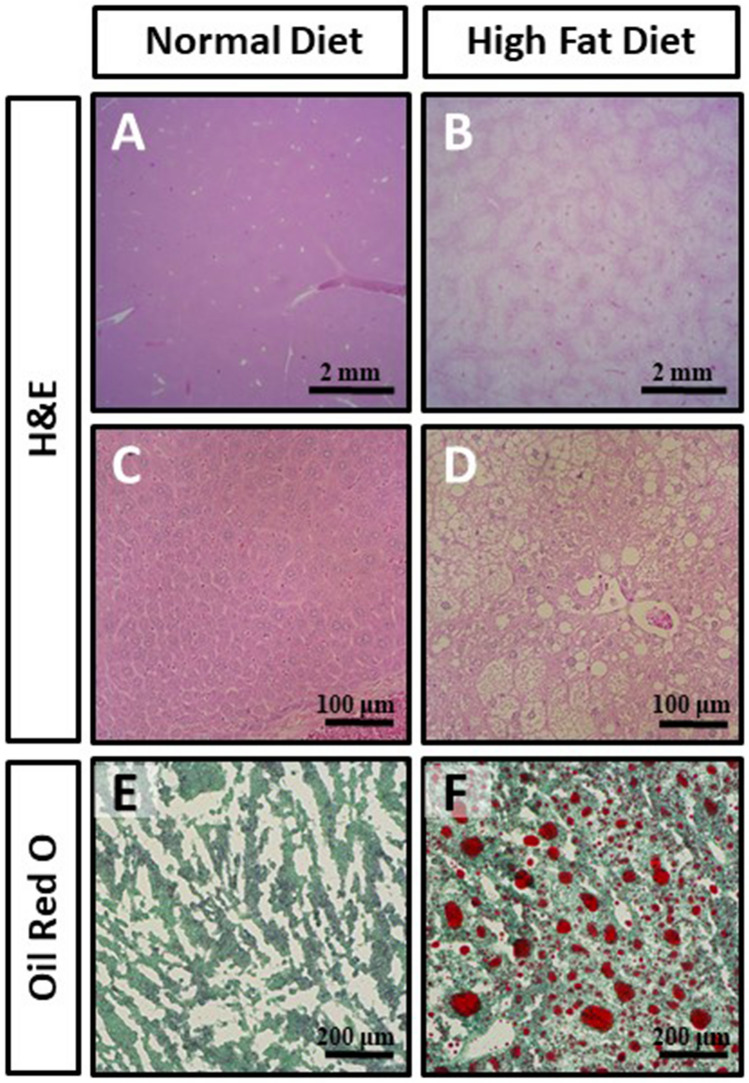
(**A**–**F**) Representative hematoxylin and eosin (**A**–**D**) or Oil Red-O stained (**E**,**F**) liver sections of mice fed a normal chow diet (**A**,**C**,**E**) or high fat diet (**B**,**D**,**F**) for 16 weeks. Images in (**A**,**B**) were taken at 1.25 × magnification, (**C**,**D**) were taken at 20 ×, while (**E**,**F**) were taken at 10 × magnification.

In order to examine the effects of obesity on cartilage health, we utilized the diet-induced obesity (DIO) mouse model. Indeed, mice fed a HFD were characterized by significant gains in body weight (Fig. 1A,B) compared to mice fed a ND (p < 0.01). In addition, glucose tolerance testing revealed HFD-fed mice had a significantly diminished ability to clear a bolus of exogenous glucose compared to mice fed a ND (Fig. 1C,D; p < 0.05). Upon gross analysis, the livers of HFD-fed mice were found to be larger and exhibit a lighter color compared to ND-fed controls (data not shown). Histological analysis confirmed the presence of atypical-appearing cells with steatotic vacuolation within the livers of HFD-fed mice compared to ND-fed controls (Fig. 2A–D). Oil Red-O staining confirmed HFD-fed mice had dramatically increased lipid droplet accumulation compared to ND-fed mice (Fig. 2E,F).

### Cartilage histology

**Figure 3:**
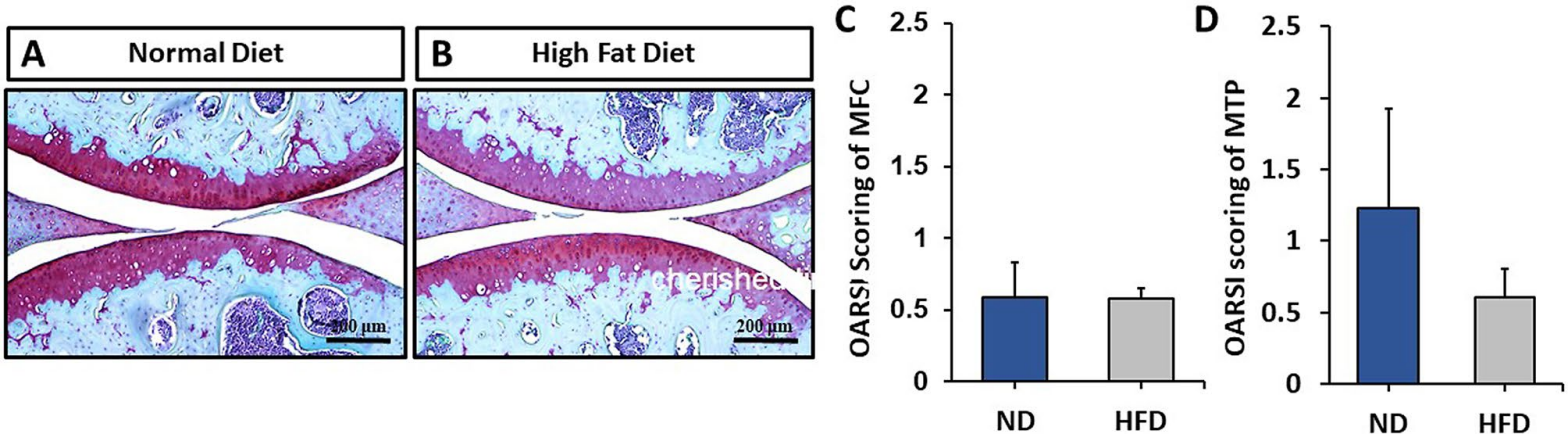
(**A**,**B)** Representative Safranin-O/Fast Green stained sections from mice fed a normal diet (ND) or a high fat diet (HFD) for 16 weeks. Images were taken at 10 × magnification. (**C**,**D)** OARSI scores of the medial femoral condyle (MFC) and the medial tibial plateau (MTP), which showed no significant effect of diet. Mean ± SEM.

**Figure 4:**
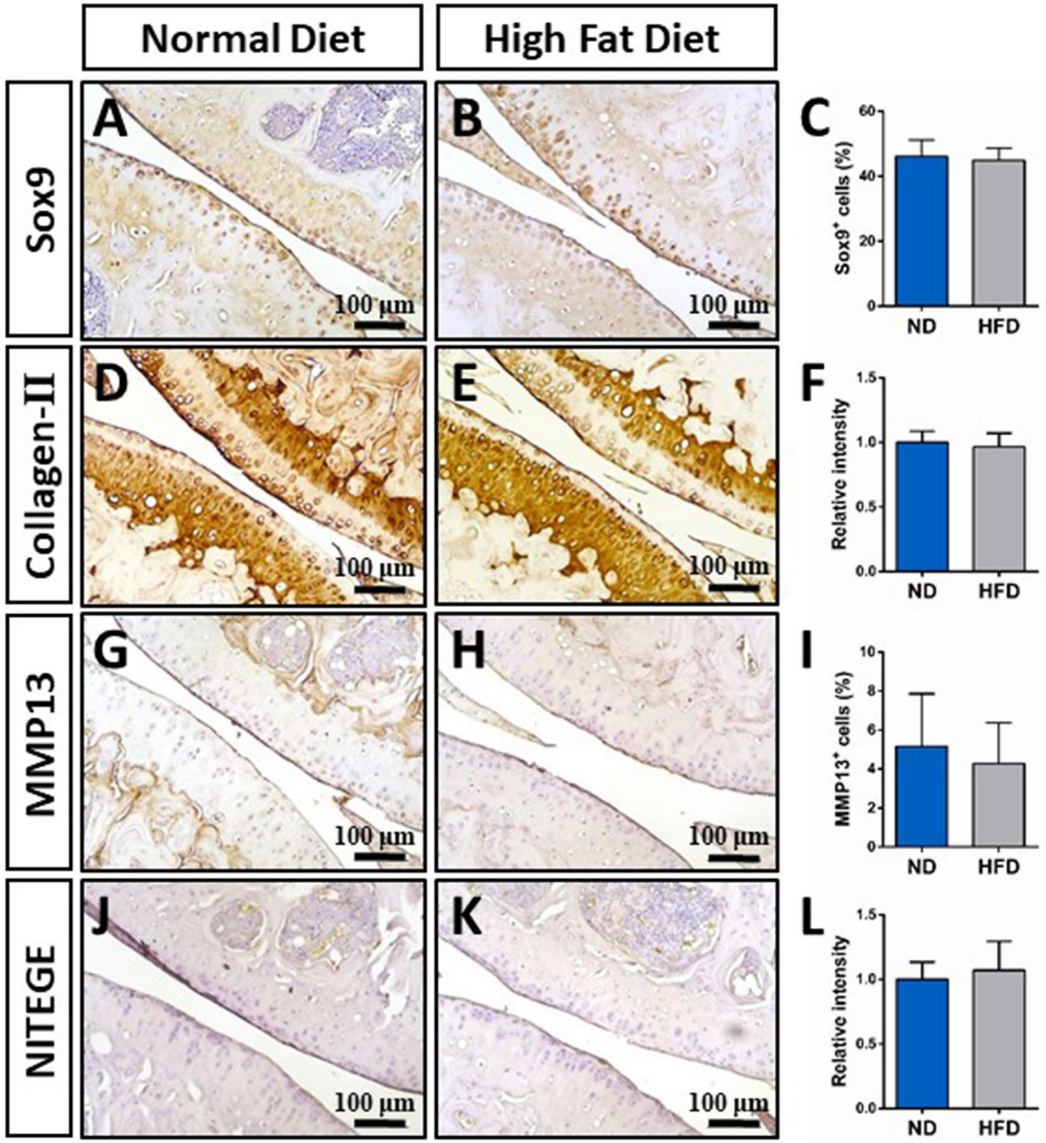
(**A**–**K**) Representative immunohistochemical analyses and corresponding barcharts evaluating the expression of SOX9 (**A**–**C**), Collagen-II (**D**–**F**), MMP13 (**G**–**I**), or the aggrecan cleavage product NITEGE (**J**–**L**) in mice fed either a normal diet or a high fat diet for 16 weeks. Images were taken at 10 × magnification. Relative staining intensity and cell quantity (%) were quantified using Image J software (Image J V1.8.0; https://imagej.nih.gov/ij/). No statistically significant differences were found between groups. Mean ± SEM.

We next sought to determine the effects of DIO on the articular cartilage of the knee. Standard histological analyses, including Safranin-O/Fast Green staining, indicated that there was no discernable difference in overall cartilage morphology or proteoglycan staining between HFD-fed mice and ND-fed controls (Fig. 3A,B). Indeed, ND-fed and HFD-fed mice had similar low-grade OARSI scores in both the medial femoral condyle (MFC) and the medial tibial plateau (MTP) (Fig. 3C,D). Further, immunohistochemical analysis found no significant differences in the expression levels of either anabolic or catabolic factors (SOX9, Collagen-II, MMP13, NITEGE) between groups (Fig. 4).

**Figure 5:**
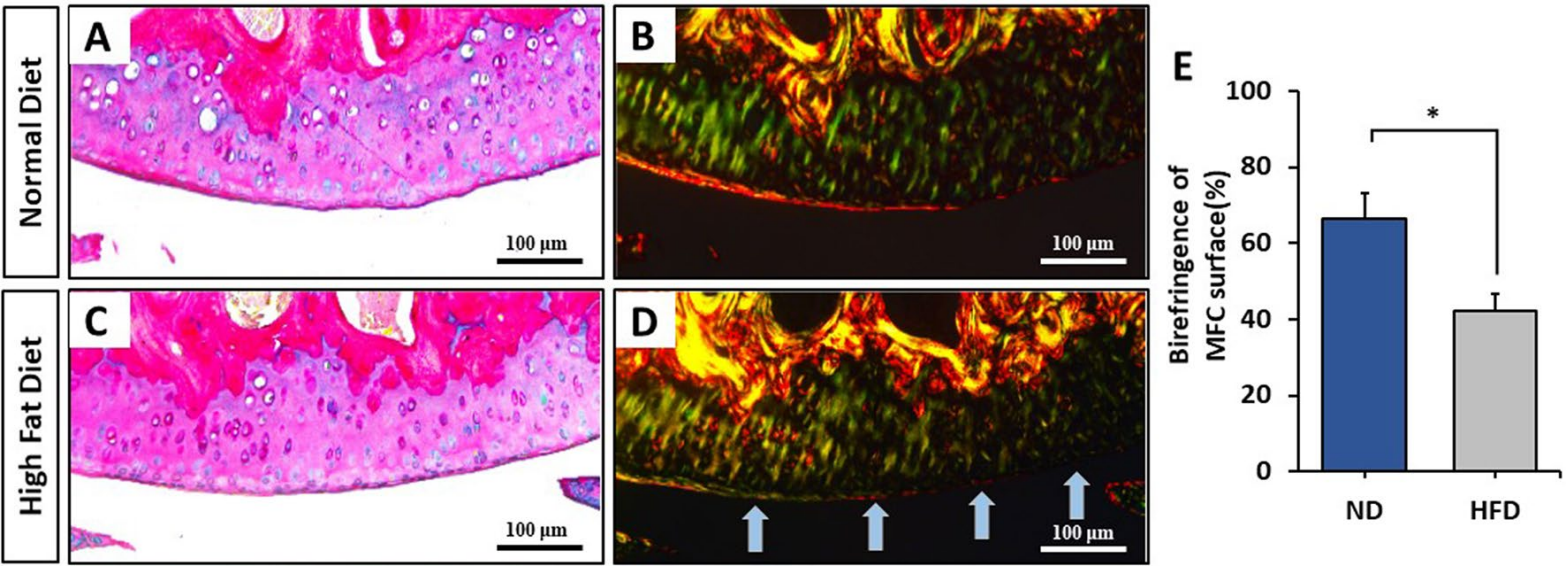
(**A**–**D**) Representative Alcian blue/Picrosirius red stained sections of mice fed a normal diet or a high fat diet for 16 weeks imaged under light microscopy (**A**,**C**) or polarized light microscopy (**B**,**D**) taken at 20 × magnification. Light blue arrows indicate loss of birefringence at the superficial layer of cartilage (**D**). Graphical depiction of the quantification of birefringence signal (**E**). Relative staining intensity and birefringence were quantified using Image J software (Image J V1.8.0; https://imagej.nih.gov/ij/). Mean ± SEM. *p < 0.05.

Polarized light microscopy of picrosirius red stained knee cartilage identified a strong birefringence signal on the superficial layer of the articular cartilage in ND-fed mice (Fig. 5A,B). This suggests the collagen in the superficial zone is well organized in ND-fed mice. In contrast, cartilage of the HFD-fed group was characterized by significantly reduced birefringence, indicative of disrupted collagen organization in the superficial cartilage layer of the MFC (Fig. 5C–E). From these data, we conclude DIO affects collagen fiber organization in the superficial layer of cartilage, which may affect cartilage biomechanical properties.

### AFM modulus results

**Figure 6:**
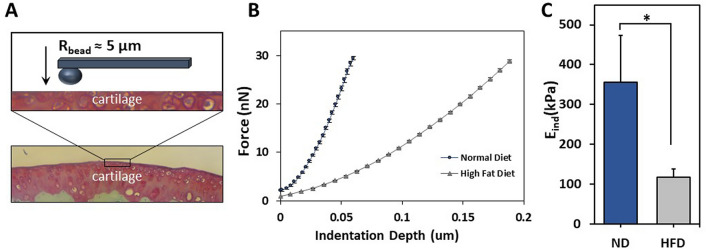
(**A**) Schematic of AFM-nanoindentation illustrating the cantilever and articular cartilage. (**B**) Representative force-indentation curves for both diet groups. Mean ± SEM (**C**) Graphical depiction of the effective indentation modulus (E_ind_) measured in mice fed a normal diet (ND) or a high fat diet (HFD) for 16 weeks. Mean ± SEM. *p < 0.05.

To determine the effects of DIO on cartilage mechanical properties, we quantified the E_ind_ using nanoindentation via AFM (Fig. 6A). The average R^2^ values for the indentation curves of the group fed a ND and the group fed a HFD were 0.998 and 0.994, respectively. Representative force-depth curves from each group are displayed in Fig. 6B, which illustrates a more compliant force-depth relationship in the cartilage of HFD-fed mice, indicating softer cartilage. To this point, E_ind_ was significantly reduced in HFD-fed mice compared to the ND-fed controls (Fig. 6C, p < 0.05). Collectively, these data indicate DIO is associated with altered mechanical properties in the articular cartilage that precedes standard histological measures of OA.

## Discussion

Here, we have determined that short-term exposure to a HFD results in alterations in the mechanical properties of cartilage, resulting in softer tissue. These changes were not associated with loss of proteoglycan as measured by standard histological scoring. Rather, HFD-induced obesity was accompanied by the disruption of collagen organization on the articular cartilage surface. These results suggest that mechanical changes may precede classical histopathological changes indicative of obesity-associated OA initiation and progression.

Recent work has demonstrated that chronic exposure to a HFD is associated with changes in cartilage histology related to OA^17^. Interestingly, in our study, acute exposure to a HFD was not associated with histological cartilage degeneration as determined by OARSI grading; however, we did observe changes in cartilage mechanical properties. This is consistent with recent studies demonstrating reductions in cartilage stiffness preceding traditional histological measures of OA in the destabilized medial meniscus (DMM) mouse model^22^. Collectively, these data indicate that mechanical changes occur early in the initiation and development of OA. Furthermore, nanoindentation of cartilage using AFM is a sensitive tool to evaluate early-stage OA^21,22,24^.

It is unclear why obesity is associated with OA risk. Initially, it was postulated to be purely biomechanical, as studies demonstrated that extreme weight loss reduced joint loading in obese adults^32–34^, thereby possibly ameliorating symptoms and slowing cartilage degradation. However, obesity has also been linked to OA-development in non-weight-bearing joints, which suggests that non-mechanical risk factors may contribute to OA development^15,34^. Interestingly, we previously reported increased cartilage strains in obese humans with no clinical indication of OA compared to humans of normal BMI^15,16^. Increased cartilage strain could be due to alterations in cartilage mechanical properties, similar to what we have observed here in DIO mice. These data suggest that altered cartilage mechanical properties may be a common consequence of obesity and an underlying factor associated with increased OA risk. However, the molecular mechanism underlying these mechanical changes is uncertain as cartilage mechanical properties are possibly affected by changes in the composition of the extracellular matrix or by changes in its structure. Indeed, HFD-fed mice showed disrupted collagen organization on their articular cartilage surface, which likely underlies the reduction in modulus (stiffness) we observed. In support of this, recent literature defined disorganized collagen fibrils and fragmentation of aggrecan in the superficial layer of cartilage as characteristic molecular events of early stage OA^35^. While the precise mechanism is not clear, increased proteolytic activity has been postulated to mediate this decline in modulus^22^. Alternatively, increased adipose tissue in obese individuals secretes cytokines that may contribute to the pathophysiological changes occurring in knee OA, including systemic and local inflammation^36,37^. Likewise, pro-inflammatory cytokines released from degradative joint tissues have been linked to reductions in proteoglycan, which is associated with reductions in the aggregate modulus of cartilage^38^.

The link between obesity and OA is likely multifactorial. Obesity may initiate and accelerate cartilage degeneration by increasing cartilage compressive forces, by increasing inflammatory cytokines that degrade the cartilage components, or by a combination of both mechanisms^39^. Here, we have demonstrated that obesity is associated with rapid alteration of cartilage mechanical properties, but the precise mechanisms of these changes have yet to be discerned. Additionally, the ability of cartilage to recover its mechanical properties is unclear; future investigations into weight loss and subsequent changes in cartilage properties are warranted.

In conclusion, ingestion of a high fat diet reduced mechanical stiffness in mouse knee cartilage. As obesity is a major risk factor in the development of OA, it is important to elucidate the role of obesity on both the temporal and molecular regulation of collagen organization and cartilage mechanics. Overall, this work highlights the importance of maintaining a healthy body weight for cartilage health and opens the door for future work investigating this important global health issue.
